# Ciguatera Fish Poisoning: Treatment, Prevention and Management

**DOI:** 10.3390/md20080022

**Published:** 2008-08-21

**Authors:** Melissa A. Friedman, Lora E. Fleming, Mercedes Fernandez, Paul Bienfang, Kathleen Schrank, Robert Dickey, Marie-Yasmine Bottein, Lorraine Backer, Ram Ayyar, Richard Weisman, Sharon Watkins, Ray Granade, Andrew Reich

**Affiliations:** 1 Mount Sinai Medical Center, Miami Beach, Florida 33140, USA; 2 NSF NIEHS Oceans and Human Health Center, Rosenstiel School of Marine and Atmospheric Sciences, Miami, FL 33136, USA; 3 Carlos Albizu University, Miami, FL 33172, USA; 4 University of Hawaii, Honolulu, HI 96822, USA; 5 University of Miami Department of Medicine/Jackson Memorial Medical Center, Miami, FL 33136, USA; 6 Food and Drug Administration, Division of Seafood Science and Technology, Center for Food Safety and Nutrition, Dauphin Island, AL 36528, USA; 7 NOAA-National Ocean Service, Center for Coastal Environmental Health and Biomolecular Research, Charleston, SC 29412, USA; 8 National Center for Environmental Health, Centers for Disease Control and Prevention, Atlanta, GA 30341, USA; 9 University of Miami Department of Neurology, Miami, FL 33136, USA; 10 Florida Poison Information Center, Miami, FL 33136, USA; 11 Aquatic Toxins Program, Division of Environmental Health, Florida Department of Health, Tallahassee, FL 32399, USA

**Keywords:** ciguatera fish poisoning, ciguatoxin, harmful algal bloom (HAB), treatment, human health, marine toxins

## Abstract

Ciguatera Fish Poisoning (CFP) is the most frequently reported seafood-toxin illness in the world, and it causes substantial physical and functional impact. It produces a myriad of gastrointestinal, neurologic and/or cardiovascular symptoms which last days to weeks, or even months. Although there are reports of symptom amelioration with some interventions (e.g. IV mannitol), the appropriate treatment for CFP remains unclear to many physicians. We review the literature on the treatments for CFP, including randomized controlled studies and anecdotal reports. The article is intended to clarify treatment options, and provide information about management and prevention of CFP, for emergency room physicians, poison control information providers, other health care providers, and patients.

## 1. Introduction

Ciguatera fish poisoning (CFP) is a foodborne illness affecting humans worldwide. Humans acquire this illness by eating reef fish containing the naturally occurring toxins, ciguatoxins. Multiple ciguatoxins have been identified, but in this paper ciguatoxins will be referred to collectively as “CTX.” CTX is derived from benthic dinoflagellates of the genus *Gambierdiscus,* growing predominantly in association with macroalgae in coral reefs in tropical and subtropical climates. The toxin is transferred through the food web as the algae is consumed by herbivorous fish, which are consumed by carnivorous fish, which are in turn consumed by humans [1–5]. This review article describes the human health aspects of CFP, as well as prevention and treatment. It also provides information about public health management of CFP particularly within the United States of America.

### 1.1. Epidemiology

The frequency of CFP varies by region throughout the world. Table 1 summarizes various regional reports on the incidence and prevalence of CFP [6].

It is estimated that 10,000–50,000 people per year who live in or visit tropical and subtropical areas suffer from CFP [7,8], but the true incidence of CFP is difficult to ascertain due to under-reporting. It is believed that only 2–10% of CFP cases are reported to health authorities [5–7,9]. In Miami-Dade County (Florida), using the degree of under-reporting from a community outbreak of waterborne gastroenteritis as a guideline, it was suggested that for every reported case of CFP, between 10 and 100 cases may go unreported [9].

Under-reporting occurs in part because individuals with CFP often do not seek medical attention, particularly in locations where CFP is well-known to local residents. However, even if they do seek medical attention, health professionals may not recognize the disease or report it to health authorities. In one study [10], a classic case of CFP was presented to 36 physicians in South Florida, where CFP is endemic. While 68% of the physicians correctly diagnosed a classic case of CFP, only about 47% of the physicians knew that CFP was a reportable disease (i.e. required by law to be reported to the health department); only 17% of the physicians recommended mannitol therapy (see below). Another factor contributing to under-reporting is whether or not a given case is part of an outbreak; a study of CFP in South Florida revealed that potential CFP cases were less likely to be reported or confirmed when a given case was a single illness, as compared to being part of an outbreak or cluster of CFP cases [11].

### 1.2. Pharmacology

CTX is one of the most potent natural substances known. One of the ciguatoxins (P-CTX-1, found in the Pacific Ocean) poses a health risk at concentrations as low as 0.08 to 0.1 μg/kg [5], although CTX rarely accumulates in fish at levels that are lethal to humans [12]. CTX activates the voltage-gated sodium channels in cell membranes, which increases sodium ion permeability and depolarizes the nerve cell. This depolarization of nerve cells is believed to cause the array of neurological signs associated with CFP [7,12–14].

### 1.3. Symptoms and Course

In the absence of a human biomarker to confirm CFP, the diagnosis of CFP is based on the clinical scenario and the patient’s recent fish-eating history. CFP is associated with gastrointestinal (GI), cardiovascular, neurological and neuropsychiatric symptoms and signs. The symptomatology has been addressed in considerable detail in numerous publications [5,12,15–20]. Table 2 summarizes the frequency of CFP symptoms in a variety of reports.

Gastrointestinal symptoms and signs (e.g. vomiting, diarrhea, abdominal pain, nausea) develop within 6–24 hours of eating a reportedly good-tasting reef fish, and usually resolve spontaneously within 1–4 days. Cardiac symptoms and signs may also occur, generally only in the early stage of the disease process; cardiac signs may include hypotension and bradycardia, and may necessitate urgent medical care.

The neurologic symptoms, which generally present within the first few days of illness, often become prominent after the GI symptoms, particularly in CFP from fish obtained in Caribbean waters. Neurologic symptoms vary among patients and include the following: paresthesias (numbness and tingling) in the extremities (feet and hands) and oral region, generalized pruritis (itching), myalgia (muscle pain), arthralgia (joint pain), and fatigue. A distinctive symptom reported by many patients is an alteration or “reversal” of hot/cold temperature perception, in which cold surfaces are perceived as hot to the patient, or produce dysesthesia (unpleasant, abnormal sensation). This temperature-related dysesthesia is considered characteristic of CFP, although not all patients report experiencing this symptom.

Temperature-related dysesthesia has been reported not only in CFP but also in Neurotoxic Shellfish Poisoning (NSP), which is caused by human consumption of shellfish contaminated by a toxin known as brevetoxin; therefore, in potential CFP patients presenting with temperature dysesthesia, if there is a history of eating shellfish then the differential diagnosis of NSP should be considered.

Of note, there is new research indicating that that brevetoxin can accumulate not only in shellfish, but also in muscles and viscera of fin-fish [21], posing a threat to marine mammals who consume them [22]. Although this suggests that theoretically, human consumption of brevetoxin-contaminated fin-fish may place individuals at risk for NSP, there are no documented cases of NSP from eating fin-fish, and the relevant concentrations of brevetoxins in fish fillets consumed as meals have not been documented at this time. Therefore, clinical presentation of temperature-related dysesthesia is still considered distinctive of CFP (rather than NSP) when there is a recent history of consuming fin-fish (as opposed to shellfish).

Neuropsychiatric symptoms in CFP may include anxiety [23], depression and subjectively reported memory loss [24]. More marked mental status changes such as hallucinations, giddiness [25], incoordination or ataxia [25,26], and coma [16] have been reported, but appear to be specific to CFP in Indian and Pacific Ocean regions.

Regional differences in symptom patterns have been noted and may be attributable to the presence of different suites of CTX or toxin precursors in different geographic regions [27]. In the Caribbean, gastrointestinal symptoms and signs predominate in the acute phase (i.e. first 12 hours [9]), with subsequent prominence of neurologic, especially peripheral neurologic symptoms. In the Pacific, the neurological symptoms and signs predominate [12] and there have been reports, though uncommon, of more severe neurologic effects including coma [16]. In the Indian Ocean, CFP has also been associated with neurological and mental status alterations, with reports of hallucinations, giddiness, incoordination, loss of equilibrium, and depression [25].

CFP is rarely fatal. However, death may occur in severe cases due to severe dehydration, cardiovascular shock during the initial illness period, or respiratory failure resulting from paralysis of the respiratory musculature [17,28], especially in areas where ventilatory support and emergency medical care are unavailable. Eating fish organs or viscera (such as the head, liver or gonads) is associated with greater symptom severity than eating only the fillet, as CTX is present in greater concentrations in such organs [29][24].

### 1.4. Chronicity

After the initial or acute illness, feelings of weakness generally last a few days to several weeks. Some patients experience chronic symptoms lasting weeks to months, in particular peripheral neurologic symptoms such as paresthesias in the extremities, pruritis, and neuropsychiatric symptoms such as malaise, depression, generalized fatigue, headaches [1,14,24,29–34].

In a small longitudinal study that assessed chronicity of symptoms in 12 CFP patients, there were no longer differences between patients and healthy control subjects on measures of neurotoxicity (e.g. paresthesias, headache, muscle weakness) or emotional symptomatology 6 months after initial illness [23]. However, the CFP cases in that study were intoxicated primarily by fish from Caribbean waters. Caribbean CTX is believed to be less acutely potent than Pacific or Indian Ocean CTX [12], and toxin potency or the regional mixture of toxins may mediate symptom chronicity.

Although rare, there are reports of neurologic symptoms, presumed to be associated with CFP, lasting for years in some patients [9,26,32]. However, such protracted complaints should be studied further to address potentially confounding psychiatric and medical explanations for them. In individual patients whose symptoms last for more than a year, explanations other than CFP should be investigated.

### 1.5. Symptom recurrence and sensitization

There are reports of sensitization to ciguatoxins in CFP patients; that is, individuals who previously suffered from CFP have been reported to experience recurrence of CFP symptoms after eating a potentially ciguateric fish that did not produce symptoms in other individuals [4,35]. Also, anecdotal reports indicate that some patients experience recurrence of neurologic CFP symptoms upon consuming alcohol, any type of fish, and certain other foods (See Table 3), even years after the initial exposure [26,36]. When this occurs many years after exposure, however, alternative etiologies for symptoms should be ruled out, given that apparent CFP symptoms and signs may actually be indicators of other severe pathology [37]. Such reports of recurrence have *not* been noted for cardiac or gastrointestinal symptoms.

One theory to explain the recurrence of neurologic symptoms is that ingested CTX may be stored in a person’s adipose tissue, and that any activity involving increased lipid metabolism may result in ciguatoxins re-entering the blood stream, with subsequent re-emergence of CFP symptoms [12]. Alternatively, symptom recurrence may be related to immunologically mediated sensitization to CTX after initial exposure [37,38].

### 1.6. Diagnosis

There are currently no reliable biomarkers that can be used to confirm exposure to CTX in humans, although studies using animals [39–41] indicate that detection of CTX in human blood or serum may be possible in the near future.

At present, therefore, CFP diagnosis is based on the presenting symptoms and time course, the history of having eaten a reef fish (as opposed to shellfish as in Neurotoxic Shellfish Poisoning), and importantly, the exclusion of other diagnoses that could account for the symptoms. CFP has symptoms in common with Paralytic and Neurotoxic Shellfish Poisonings, scombroid and pufferfish toxicity, botulism, enterovirus 71, and bacteremia [7,37], as well as organophosphate pesticide poisoning, eosinophilic meningitis, multiple sclerosis, and other neurologic conditions.

The current gold standard diagnosis of CFP would include confirmation of CTX in the consumed fish by appropriate laboratory methods (see Detection in Fish, below). Also, multiple individuals consuming the same fish, with all individuals experiencing signs, symptoms and time course consistent with CFP, strongly supports the CFP diagnosis [42].

### 1.7. Detection in Fish

Although diagnostic methods for detecting CTX in human specimens are still under development, methods for detecting the toxins in samples of the implicated fish have been developed, and the results have been used to support clinical diagnoses of CFP in the United States, Caribbean and South Pacific. Within the USA, laboratories of the Food and Drug Administration (FDA) perform CTX analyses on fish to assist in the CFP diagnosis of patients. Remnants of consumed fish can be submitted to the FDA for analysis, (see Recommendations, below). However, because the results of such analyses are generally not immediately available to the physician at the time of the patient’s initial presentation and examination, initial care of the patient must proceed based on symptom progression, recent fish-eating history, and exclusion of alternative diagnoses or explanations.

The FDA fish testing procedure is a two-tiered protocol involving: 1) *in vitro* assay, i.e. a high-throughput screen for toxicity consistent with ciguatoxin’s mode of action; and 2) an analytical chemistry technique known as liquid chromatography-mass spectrometry (LC-MS).

In the first tier, suspect fish specimens are screened for the specific effects of CTX. Specifically, fish specimens are screened for voltage-gated sodium channel–specific activating effects on cell membranes. The screening procedure used is the *in vitro* mouse neuroblastoma (N2a) cell assay (American Type Culture Collection CCL-131), using a ouabain-veratridine dependent method [43,44]. Through appropriate experimental design, this screening method can detect effects of algal toxins on sodium channels in cell membranes, can discriminate between effects of toxins that activate (e.g. brevetoxins, ciguatoxins) versus block (e.g. tetrodotoxins, saxitoxins) voltage-gated sodium channels, and can discriminate between the effects of sodium channel specific toxins and toxins with other modes of action. However, this screening procedure does not identify specifically the sodium channel active agent present in the fish tissue. Therefore, in the second tier, fish samples that test positive for sodium channel specific toxicity are subjected to LC-MS, to confirm the molecular presence of CTX in the fish tissue. Multiple Reaction Monitoring (MRM) is used to selectively detect product ions from ciguatoxin precursor ions. This method provides a high level of specificity for detecting CTX in fish [45].

Of note, alternative methods for CTX detection in fish tissues with equivalent or lesser sensitivity and specificity have been reported, and include receptor binding assay which is also sodium channel specific and is considered to be sensitive [46–51]; LC-MS methods [48,52,53]; and a host of other less frequently employed assay systems (see review by Lewis, 2004 [54]).

While the fish analysis procedures described here are performed generally in specially equipped laboratories by trained professionals, attempts have been made to develop simpler procedures that are cost-effective and can be used to detect ciguatera toxins on a routine basis by fishermen, fish vendors or consumers. However, developing such simplified techniques involves substantial challenges, including the low levels of CTX present in ciguateric fish (<0.05 parts per billion for one common type of CTX molecule), multiple structural toxin forms present within a single fish, and the small quantities of ciguatera compounds available for research [27]. A membrane immunobead assay was developed in Hawaii [55] as a simplified ciguatoxin detection procedure, but available studies evaluating the immunobead procedure on samples from the Caribbean and from Hong Kong revealed false negative detection rates ranging from 50–82%, and false positive rates ranging from 33–44% [56,57]. To date, there is no commercially available fish-testing product that has been demonstrated by independent investigation to provide ciguatoxin detection with adequate accuracy [56,57].

Access to CTX for research purposes is a significant challenge. Purified CTX from the Caribbean and Pacific are not widely available for laboratory experimentation due to the low amount of toxin present in fish, although a few specialized laboratories have purified sufficient quantities for research and outbreak investigations [e.g. Murata *et al.* 1989, 1990 [59,60]; Crouch *et al.* 1995 [61]; Vernoux *et al.* 1997 [62]; Lewis et al, 1991 [63]; Hamilton *et al.*, 2002] [53].

## 2. Treatments

Over the years, a myriad of treatments, mostly responding to CFP symptoms, have been tried. However, there have been few well-designed randomized controlled trials investigating the effectiveness of specific treatments for CFP. Some of the research design challenges and problems include the relative infrequency and irregularity of CFP’s occurrence, lack of resources for conducting scientific treatment investigations in those places where it occurs with greater frequency, the inability to confirm the diagnosis of CFP using a human biological assay, small numbers of patients (which is particularly problematic when attempting to compare subgroups of participants), variability among study participants in time to treatment, and potential differences between Pacific, Indian Ocean, and Caribbean toxins and their effects. In addition, in the CFP literature, unless fish testing was available, it is generally unknown if the case reports and even clinical trials of CFP treatments were treating actual cases of CFP.

### 2.1. Mannitol

Intravenous (IV) mannitol infusion is the most studied therapy for CFP, and the only therapy evaluated by randomized blinded clinical trials [64,65]. IV mannitol is administered at 0.5 to 1.0 g/kg body weight over a 30–45 minute period. It is suggested that it be given within 48–72 hours of ingestion of toxic fish [66,67], although beneficial effects have been observed even up to several weeks after intoxication [32,68]. The effect of mannitol infusion is thought to be mediated by the osmotic reduction of neuronal edema [1]. Also, mannitol may act as a scavenger of free radicals generated by the CTX molecule, and may reduce the action of CTX at sodium and/or potassium channels [69]. Nicholson and Lewis note that hazards of mannitol’s clinical use include loss of further fluids in patients suffering from acute diarrhea and vomiting, and that patients experiencing bradycardia and hypotension are at higher risk of cardiac failure if infused with high doses of mannitol [12]. Lewis and King [70] indicated that mannitol should not be administered until the patient is adequately rehydrated. Mannitol therapy has been recommended for two primary goals: Reduction of acute symptoms (especially neurologic) and possible prevention of chronic neurologic symptoms.

Early descriptive reports on the use of IV mannitol suggested that it provided significant, rapid improvement and even resolution of acute CFP signs and symptoms. Also, based on the anecdotal reports that individuals treated with IV mannitol appeared to show reduced likelihood of returning for additional medical care, it was suggested that IV mannitol may even prevent the development of chronic CFP symptoms [32]. Palafox *et al.* [67] described 24 cases, 17 of whom experienced complete resolution of CFP symptoms within 48 hours of infusion, including two cases of coma and one case of shock who improved within minutes of infusion. They suggested that treatment with IV mannitol was associated with shortened time of significant morbidity and shortened hospital stays, based on their clinical experience. Another early report [71] examined 12 CFP cases, and indicated that IV mannitol was generally effective at alleviating symptoms when administered within several days of fish ingestion. Notably, in this report, one case showed complete recovery by 24 hours after ingestion without any treatment.

Blythe *et al.* [68] provided a descriptive report on the experience of local South Florida practitioners using IV mannitol to treat patients with acute and chronic CFP symptoms. The report described 70 CFP cases treated with IV mannitol; the mean time from exposure to clinical presentation was 11.5 days (SD=44.5; range of 0.3 to 365 days). The study indicated that 29 of the 32 (91%) patients who received mannitol within 48 hours of presumed exposure experienced complete reversal of symptoms. In addition, IV mannitol was associated with moderate reduction, and in some patients complete resolution, of symptoms if given from 3–14 days after exposure; there was also a reported benefit when administered up to 70 days after exposure. Multiple treatments (e.g., up to four additional infusions) were necessary in five individuals. The authors reported that there were no adverse effects of mannitol treatment. Although 37 non-mannitol patients were included in the case series, analysis comparing symptom outcome between mannitol and non-mannitol treated patients was not reported, and there was no randomization or blinding employed as part of the study design, so it cannot be ruled out that patient or interviewer expectations, rather than the mannitol itself, accounted for all or part of the reported benefit in patients who received mannitol.

A study by Bagnis *et al.* [65] randomized 63 CFP patients in French Polynesia to one of two treatment groups: IV mannitol infusion (n=34), or “standard” treatment (n=29) which included intravenous infusion of glucose, Vitamin B and calcium gluconate. Results indicated a statistically significant, greater reduction in symptomatology in the mannitol group compared to the standard treatment group.

Schnorf *et al.* [64] conducted a double-blind, randomized study of mannitol treatment. Fifty patients were included in the study and were randomized to either mannitol treatment (n=25) or normal saline infusion (n=25). Results indicated that both treatments were associated with clinical improvement and to a similar degree, and mannitol was not superior to saline. However, 25% of the mannitol group received their mannitol between 69 hours and 672 hours (28 days) after symptom onset, which is generally beyond the 48–72 hour period recommended by other reports [32,66,68], and which may have obscured the identification of benefits in patients who received it early on. The authors then compared outcome of patients receiving mannitol within 24 hours to those receiving it after 24 hours, and found no difference. However this subpopulation analysis suffered from small sample size. In addition, by truncating the group at 24 hours post symptom onset rather than 48–72 hours (which has been associated with mannitol benefit [32,66,68]), true difference in benefits may have been diluted in the comparison. Also, as is common in CFP treatment studies, the results may have been confounded by potential CFP misdiagnoses among study participants, since the diagnosis of CFP cannot be confirmed by any tests using human fluid or tissue samples, and since the study did not employ fish testing to confirm CTX in remnants of fish consumed by patients.

### 2.2. Symptomatic and Supportive Treatments

With acute CFP, support of any depressed vital functions is of utmost importance [70], and supportive therapies may be necessary for controlling fluid and electrolyte balance [27]. As in any acute poisoning, IV fluid resuscitation with large volumes of isotonic fluids may be necessary for patients in shock, with the addition of an IV pressor infusion if needed after volume repletion. Symptomatic bradycardia may require IV atropine dosing as needed (0.5 mg every 5 minutes to maintain a heart rate goal of 60 beats per minute, with no maximum total dosage limit) and/or temporary cardiac pacing. Rarely, critically ill CFP patients may require endotracheal intubation and mechanical ventilation for either airway protection if comatose or for respiratory failure. The prognosis for full recovery of critically ill patients is excellent with intensive care treatment when needed. Also, patients seen within the first few hours after ingestion of toxic fish may benefit from treatment with oral activated charcoal to prevent further absorption of toxin from the gut, although ongoing severe vomiting and diarrhea may prevent this [7].

Various medical treatments for other CFP symptoms have been tried, but with variable success [72] and without randomized controlled trials to establish their effectiveness for this purpose. For instance, fluoxetine has been used for chronic fatigue [73]; amitriptyline for paresthesias, pruritis and headaches [74], [75]; and paracetemol (acetaminophen) and nifedipine for headaches [20,73]. Gabapentin has been used to treat pain [76] but caution is warranted in prescribing medications with addiction potential, given that there are no randomized controlled trials examining their safety and effectiveness for CFP, that patients’ complaints may last for months, and that chronic complaints may be confounded with psychiatric or other medical causes. It has been recommended that opiates and barbiturates be avoided since they may lead to hypotension, and because opiates may interact with maitotoxin, a natural marine toxin that may also be present in ciguatoxic fish [6].

There is also a variety of traditional herbal medicines and remedies that have been used to treat ciguatera. For instance, 64 different plant species have been reportedly used in the Western Pacific [77], and extracts of *Argusia argentea* leaves or *Davalliea sp*. have been reported in New Caledonia [33]. However, there is no scientific evidence demonstrating that these remedies are safe or effective in treating CFP [12].

### 2.3. Food and Dehydration Avoidance

Frequent anecdotal reports to physicians and investigators indicate that after experiencing CFP, ingesting alcohol [4,6,26,27,74] and any kind of fish [4,6,26,27,35] can cause an augmentation or relapse of symptoms. Ingesting caffeine [6], nuts [6,8], chicken [4,8] and pork [8], or experiencing physical over-exertion or dehydration[74] have also been associated with symptom recurrence or augmentation. Therefore, patients are advised to avoid behaviors or activities that cause dehydration, and to avoid such foods (Table 3) for 3–6 months after intoxication or until all CFP-related symptoms have resolved. However, controlled investigations of the incidence of symptom recurrence as well as the effectiveness of specific food and activity avoidance measures have not been conducted, and any beneficial impact may vary among individuals.

## 3. Prevention

CFP is difficult to prevent because CTX in fish is odorless and tasteless, and toxic fish cannot be identified by appearance or behavior. CFP occurrences are not attributable to inadequate food handling, storage, preparation, or procurement methods for the contaminated fish. CTX is heat-stable, and therefore, cooking, boiling, freezing, baking or frying does not eliminate or destroy the toxin from the fish tissue [28]. Person-to-person transmission of CFP is exceedingly rare, but there are reports of transmission of GI symptoms from an acutely ill mother across the placenta to the fetus/newborn [6] and to a nursing infant [78], and of transient genital paresthesias in sexual partners of those with acute CFP [79].

### 3.1. Avoiding Ciguateric Fish

Prevention of CFP relies on the individual’s avoidance of fish that have a greater likelihood of ciguatoxicity. Local residents in endemic areas often report being aware of specific reefs and/or seasons to avoid when fishing. Local residents also report a variety of simple home tests to detect toxic fish, but none of these are scientifically validated.

Intoxication is associated with consumption of large reef fish, and greater illness severity is associated with eating the fish viscera and larger portions [70]. Thus, people are advised to avoid eating the viscera of reef fish. It is also advisable to avoid consuming large predatory reef fish from areas known to be associated with CFP. With regard to fish size, avoidance of fish in excess of 3 kilograms (approximately 6.6 pounds) [37], or even more conservatively, avoidance of fish in excess of 1.35 kilograms (approximately 3 pounds) [80] has been recommended. It has also been proposed that eating small portions (i.e. <50 grams or < 0.11 pounds) of different fish is safer than eating larger portions of any individual fish that might be associated with CFP [27,70].

Table 4 provides a list of commonly ciguatoxic fish to avoid [4,32,52,81–84], although any large coral reef fish has the potential to be ciguatoxic. Of note, recent reports [85] suggest that CFP may be associated not only with coral reefs, but also with oil rigs and other artificial reefs.

In laboratory studies, fish that ingested ciguatoxic barracuda or freeze-dried cells of *Gambierdiscus toxicus* (i.e. the ciguatoxin-producing dinoflagellate) displayed skin color variation, inactivity, erratic swimming, and even death above certain dosages [86,87][74,75]. However, toxic fish in the field generally do not appear to be harmed by CTX accumulated in their tissue; there is no reliable method of detecting ciguatoxicity in fish in the field based on their appearance or behavior.

### 3.2. Surveillance and Reporting

CFP is reportable to the health authorities in various states in the USA. For example, in the state of Florida, licensed physicians, chiropractors, naturopathy practitioners, and veterinarians are required by law to report suspected cases of CFP to the Department of Health (DOH) (Section 381.0031(1,2), Florida Statutes). Suspected cases are then investigated by public health officials and confirmed cases are recorded in the state’s registry of reportable/notifiable diseases. Investigation of cases includes a telephone or face to face interview to gather case details, collection of remnants of the consumed fish for shipment for analysis at the FDA, and collection of medical and laboratory data needed to confirm the diagnosis and aid in surveillance. Determining the source of the fish purchased or place of capture is important in preventing additional cases that may occur when a large, recreationally caught fish is shared or when a wholesaler may have shipped toxic fish to other areas for resale.

The Florida DOH also maintains a Food and Waterborne Disease Surveillance Program with a website providing information on CFP and other food borne diseases. This program contains a network of regional epidemiologists which coordinate with county health departments, the Florida Poison Information Center (FPIC), emergency departments and other health care providers to facilitate case investigation, reporting and public health education (www.myfloridaeh.com).

The Florida Poison Information Center- Miami (FPIC-M) maintains a toll-free Aquatic Toxins Hotline which is available 24 hour/day (888-232-8635; from outside of the US but not toll-free: 305-585-5250). The FPIC-M collects information on CFP occurrences and provides a summary of this data to the Florida Department of Health (DOH). The Florida DOH uses this information to augment surveillance of CFP and other marine and freshwater toxin illnesses for the Florida DOH and the Centers for Disease Control and Prevention. The FPIC-M also provides diagnostic, treatment, and educational information to callers from any location.

In the USA, the FDA’ s Center for Food Safety and Nutrition (CFSAN) conducts research on CFP and collaborates with academic and government counterparts, as part of its mission to protect and advance public health. The FDA has developed methods to screen and confirm the ciguatoxicity of CFP-implicated fish remnants, and collects data on ciguatoxic fish species, toxin concentrations associated with human illness, and potential biomarkers of CFP in human body fluids. Therefore, submitting fish remnants to the FDA for CTX analysis contributes data essential for improving estimates of the true incidence of CFP, and for developing regulatory guidelines to reduce the incidence. For patients whose fish remnants are submitted to the FDA, current research efforts include the collection of patients’ blood and urine samples, in order to contribute to the development of a human *biomarker* of CFP, to assist in clinical diagnosis.

Of note, in the USA, the National Oceanic and Atmospheric Administration (NOAA) Centers for Coastal Ocean Sciences (NCCOS) also conducts research on ciguatoxicity of fish as part of its mission to provide scientific information that addresses society’s environmental, economic and social goals, and promotes the health of the ecosystem and natural resources.

### 3.3. Education and Outreach

Community outreach and education on CFP are needed, given the under- and misdiagnosis, as well as under-reporting of it [88–93]. Primary prevention of CFP relies heavily on providing education about CFP and the fish associated with it to consumers, especially those most susceptible to CFP, such as recreational and subsistence fishermen and the communities that they serve. Additionally, identifying local reefs with a high density of ciguatoxic organisms and/or fish, and communicating this to fishermen via posting and other announcements is important, to help prevent ciguatoxic fish from entering the fish market.

**Figure 1:**
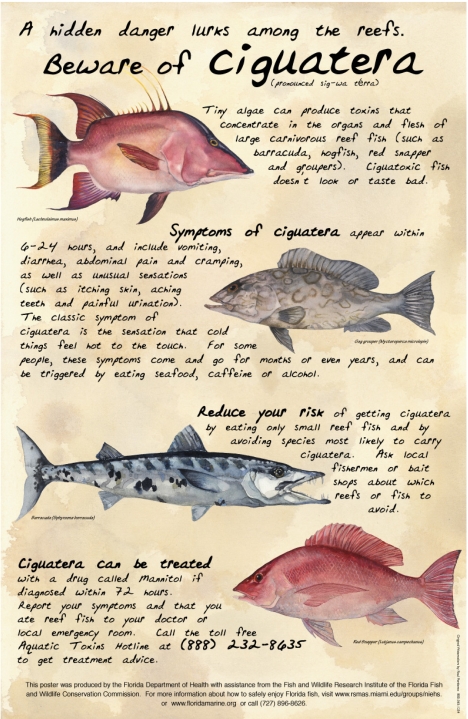
Example of outreach and educational materials developed for CFP (http://www.doh.state.fl.us/environment/community/aquatic/index.html); this Florida poster is targeted at fishers (W.B. Stephan MPH, Health Educator, FPIC-M)

A number of health departments, other government agencies, researchers and grassroots groups (particularly in the diving community) have produced a range of materials of varying quality concerning various aspects of CFP. An example of high quality information is the information provided by the Florida Department of Health Aquatic Toxins Program which maintains a website with downloadable materials for a range of target audiences on CFP and other marine and freshwater toxin diseases (Figure 1) (http://www.doh.state.fl.us/environment/community/aquatic/index.html).

As noted above, the FPIC-M maintains a toll free 24-hour hotline with information on CFP, and provides the opportunity to speak directly to a trained Poison Information Specialist [93].

## 4. Future Directions

A number of future developments may help reduce the occurrence and impact of CFP in humans. First, to improve assessments of the true incidence of CFP, it is important to increase awareness and reporting of CFP occurrences to local health departments. Making CFP a “reportable” or “notifiable” disease in as many states as possible, even states where ciguatoxic fish are not typically caught, would increase awareness of the disease and facilitate better estimates of incidence; this is especially important given that ciguatoxic fish may be shipped to, resold, and ultimately consumed in a state other than the state where it was originally caught. Also, obtaining professional validation of the ciguatoxicity of suspect fish remnants is important for improving assessments of the true incidence of CFP.

At the point of fish sale, or when consuming a pre-cut or pre-packaged portion or a prepared fish meal (e.g., a fish stew), individuals may not know the geographic origin, size or type of fish they purchase either for resale or for consumption. Therefore, tagging and tracking the geographic origin (including latitude and longitude data), size and type of fish, and making such information available to vendors and consumers may improve their ability to avoid ciguatoxic fish. Alternatively, bans may be placed on the capture or trade of certain fish species or fish from certain locations, or on coral reef fish over a certain size [27,42]. Posting announcements to fishers indicating which reefs are associated with known ciguatoxic fish may help to reduce CFP occurrences.

One of the most important aspects of future management of CFP is the development of simple, cost-effective methods of detecting ciguatera toxins in fish, which can be used by fishers, vendors and consumers, and which can provide satisfactory levels of accuracy. Research involving antibodies and reagents to synthesized CTX shows considerable promise for the future [94]. In humans, the development of methods to confirm exposure to CTX in affected individuals will assist not only in the diagnostic process, but in the validity of clinical trials investigating treatments for individuals with a presumed CFP diagnosis.

With regard to treatment, one future direction pertains to brevenal, a recently discovered antagonist to brevetoxins (the toxins associated with the dinoflagellate, *Karenia brevis*, or the Florida red tide). Brevenal has been patented for possible use in brevetoxicosis in animals and humans. Due to the structural and physiologic similarity of brevetoxins and ciguatoxins, in the future, it is possible that brevenal (and the synthetic beta naphthoyl brevetoxin) may be used as a treatment for acute CFP in humans (Abraham et al., 2005 [95]; personal communication, LEF and Dr Daniel Baden, UNC Wilmington, NC). More generally, future randomized, double-blinded controlled studies are needed to provide additional evidence to support or negate specific treatments for CFP, and to determine the best timing of treatment in the course of the illness.

## 5. Recommendations

Based upon available evidence and anecdotal experience, the authors recommend the following “best practice” guidelines for CFP clinicians:

1. Use of IV mannitol: If a patient meets the criteria for a diagnosis of CFP, consumed the implicated species within the past 48–72 hours, and there are no contra-indications to its use, then treatment with IV mannitol is recommended. Repeat treatments may be necessary if symptoms return. After 72 hours, IV mannitol treatment may be considered on a case by case basis, though it is not routinely recommended. Prior to treatment, as part of the process of informing patients about treatment options, patients should be informed about the inconsistency of research findings on the effectiveness of IV mannitol for CFP, the limitations of knowledge about its effects, as well as the fact that insurance will often not pay for this treatment. In all cases, the decision to proceed with mannitol treatment should be based upon the risk-benefit analysis, discussing such risks and benefits with the patient, and ultimately, the personal preference of the patient after being informed that it may not work.
2. Supportive and symptomatic medical treatments: Supportive and symptomatic medical treatments for CFP symptoms should be determined on a case by case basis, according to the patient’s health situation, with the caveat that there are no randomized controlled studies investigating the effectiveness of any medical treatments other than mannitol for CFP. Caution is warranted in the use of medications with addictive potential for treating CFP symptoms, especially given the lack of research evidence supporting the safety or effectiveness of such medications for that purpose.
3. For patients with chronic complaints that are ***not*** clearly caused by CFP, it is recommended that they receive a full evaluation by a neurologist, internist, and psychologist and/or psychiatrist, who can provide joint input on the diagnosis and a recommended plan of care.
4. For patients with chronic complaints that ***do*** appear to be caused by CFP, based on experience at a local South Florida care center, patients may benefit from a low dose of selective serotonin reuptake inhibitor (SSRI), as well as a combination of assessment and care including a physical/occupational therapist, psychologist, and group psychotherapy sessions and/or family counseling, as needed, on a case by case basis.
5. Fish avoidance: Avoidance of fish reported to be associated with CFP, as well as any large reef fish, large fish portions, head and viscera (e.g. liver, gonads), and fish obtained from regions known to be associated with CFP, is recommended (Table 4).
6. Avoiding symptom recurrence: Avoidance of dehydration and certain foods is recommended for 3–6 months after initial CFP intoxication or until the patient is symptom-free (Table 3). As an alternative to food and alcohol avoidance, patients may opt to try certain of the listed items but should be advised to do so cautiously and watch for recurrent symptoms. These recommendations are made with the following caveats: a) This is based on anecdotal reports; b) There are no empirical studies verifying the utility of such interventions, and c) This may not be helpful to all CFP patients.
7. Report CFP: CFP should be reported to state or local health authorities. In some states (e.g. Florida), licensed physicians, laboratories and certain other practitioners are required by law to report suspected cases of CFP. Also, the Aquatic Toxins Hotline of the Florida Poison Information Center in Miami is a 24-hour resource available to people nationwide (888-232-8635; from outside of the US but not toll-free: 305-585-5250).
8. Submit fish and clinical samples for testing: Upon determining that a patient has an illness caused by seafood consumption, physicians are encouraged to record time of meal consumption, amount of time between meal consumption and symptom onset, and obtain a detailed profile of patient symptoms. Physicians, other health care providers, or public health officials should also request from patients remnants of the fish that was actually consumed. Obtaining related information about the fish such as the kind of fish (e.g. grouper, snapper, amberjack, *etc.*), where it was purchased or caught, and other people consuming the same fish (symptomatic or not) is also helpful.
  In the USA, implicated remnants of the consumed fish and the case-related information should be sent to the FDA laboratory for analysis of ciguatoxicity by public health authorities or poison control centers responding to the report, or by the Emergency Department physicians. In addition, in cases where fish remnants are being submitted for analysis, clinical samples of the patient’s urine and whole blood, taken as close as possible to the time of symptom onset, may be solicited for submission to the FDA. For instructions on submitting fish and clinical samples, the FDA contact person is Ray Granade (*hudson.granade@fda.hhs.gov*), phone: (251) 690–3379.
9. Randomized clinical trials of sufficient size are needed to document the occurrence of CFP, and the efficacy of IV mannitol and other common treatments.
10. Creation of messages is recommended, to inform the public, fishermen, fish vendors, and restaurants about where to obtain good information on ciguatera.
11. Educational modules targeted at medical students, emergency department personnel, poison information center personnel, and other health care providers should be developed and implemented.

## Figures and Tables

**Table 1:** Reported Incidence and Prevalence of CFP.

Geographic Region	Incidence/10,000/year	Data collection time period	Reference
Reunion Island	0.78	1986–1994	Quod 1996 [25]
Queensland, Australia	3	1965–1984	Gillespie 1986 [4]
Hawaii	0.3	1975–1981	Anderson 1983 [96]
US Virgin Islands	7.6	1982	Morris 1982 [97]
Guadeloupe	30	1984	Czernichow 1984 [98]
South Pacific Region	970	1973–1983	Lewis 1986 [99]
Marshall Islands	2,820	1982–1983	Lewis 1986 [99]
French Polynesia	5,850	1979–1983	Lewis 1986 [99]
Dade County, FL	5	1974–1976	Lawrence 1980
Culebra, Puerto Rico	73.6–169.5	2005–2006	Luber, In prep [100]
Geographic Region	Prevalence (%)	Time range	Citation

St. Thomas (US Virgin Islands)	4.4	Annual (1980)	McMillan 1980 [101]
Puerto Rico	7	Lifetime	Holt 1984 [102]
Tahiti	8.45	Annual (1966)	Bagnis 1979 [16]
Hao (Tuamotos)	43	Annual (1978)	Lewis 1986 [99]
Polynesian Islands	70	Lifetime	Lewis 1986 [99]

**Table 2:** Reported Frequency (%) of Clinical Symptoms of Ciguatera at Time of Diagnosis

	Region of Study Author Number of Cases												
Oceans	Caribbean						Atlantic	Pacific					Indian
Reported Symptoms	Friedman [23]	Arena [24]	Stinn [42]	Frennette [103]	Engleberg [104]	Escalona [105]	Lawrence [9]	Bagnis [26]	Schnorf [64]	Chateau-Degat [106]	Gillespie [4]	Bagnis [16]	Quod [25]
	N=12	N=12	N=442	N=57	N=47	N=80	N=129	N=12,890	N=50	N=47	N=527	N=3009	N=167
**Gastrointestinal:**													
Diarrhea	67	75	79	77	81	83	76	73	50		64	71	49
Vomiting		42	43	37	40	69	68	39			35	38	50
Nausea	42			82		69		44	26		55	43	50
Abdominal Pain	42	75	65	58	30	74		43	52		52	46	29
**Neurologic:**													
Extremity Paresthesia	67	100	81	79		36	71	89	72	93	64–71	89	82
Circumoral	58		70	79	38	38	54	88		91	66	89	82
Paresthesia													
Temperature	58	92	64	77	23	48		87	94	34	76	88	65
Dysesthesia													
Myalgia	67	75	79	75	34	56	86	85	56	80	83	82	38
Arthralgia	42	83	79	75	34	60		86	62	80	79	86	29
Pruritis	67	67	77		66	45	48	44	42		76	45	5
Headache				56	45	39	47	60	50		62	59	25
Vertigo	25	58	50			33	47		62		45	42	
Weakness (Asthenia)	92	100		84		65	30	60	80			60	70
Dental Pain/Feeling like teeth are loose or falling out	33		32	23	13	11		21			37	25	
Dysuria	8	33	25					13	26		22	19	
Chills/Sweating					36		24	60			49	59	
**Neuropsychiatric:**													
Hallucinations	8	17										<5	16
Depression	25	17											16
Memory/concentration problems	17	58											
Multi-tasking problems	25												
Giddiness								29					30
**Cardiovascular:**													
Arrhythmia	33												
Hypertension								12	12				
Bradycardia								16	16				

Notes: Blank cells indicate that data on that symptom were not reported in the study referenced.

The table does not provide relative risk data, i.e. it does not provide comparative information on symptom frequency in an unexposed population. Table modified from Stinn *et al.*, 2000; Arena *et al.*, 2004

**Table 3:** Foods and behaviors associated with symptom recurrence.

Alcohol [4,6,27,74]
Nuts [6,8]
Caffeine [6]
Pork [8]
Chicken [4,8]
Any kind of fish [4,6,27]
Physical activity/exertion [74]

**Table 4:** Fish avoidance recommendations.

Some Common Ciguatoxic Fish [4,32,52,81–84]
Moray eel
Barracuda
Grouper
Kingfish
Jacks
Snapper
Surgeonfish
Parrot fish
Wrasses
Hogfish
Narrow barred Spanish mackerel
Coral trout
Flowery cod
Red emperor
The following are also associated with CFP
Eating fish viscera or roe [29,70]
Large, predatory reef fish* [37,70,80]
Reef fish from areas known to be associated with CFP occurrence [70]
Note that eating small portions (i.e. <50 grams or <0.11 pounds) of different fish may be safer [27] than eating larger portions (i.e. >200 grams) of any one potentially ciguatoxic fish [70].

* Lange *et al.*, 1987 cites a recommendation to not eat fish larger than 1.35–2.25 kg (3–5 pounds), whereas Ting and Brown (2001) recommend not eating fish larger than 3 kg (6.6 pounds).
